# First isolation and molecular characterization of canine parvovirus-type 2b (CPV-2b) from red foxes (*Vulpes vulpes*) living in the wild habitat of Turkey

**DOI:** 10.1186/s12985-023-01988-2

**Published:** 2023-02-11

**Authors:** Hanne Nur Kurucay, Cuneyt Tamer, Bahadir Muftuoglu, Ahmed Eisa Elhag, Seda Gozel, Yasemin Cicek-Yildiz, Sadik Demirtas, Emre Ozan, Harun Albayrak, Semra Okur-Gumusova, Zafer Yazici

**Affiliations:** 1 Department of Veterinary Virology, Faculty of Veterinary Medicine, Ondokuz Mayis University, 55139 Atakum, Samsun Turkey; 2 Department of Veterinary Experimental Animals, Faculty of Veterinary Medicine, Ondokuz Mayis University, 55139 Atakum, Samsun Turkey; 3 Department of Preventive Medicine and Clinical Studies, Faculty of Veterinary Sciences, University of Gadarif, 32211 Al Qadarif, Sudan; 4 Samsun Veterinary Control Institute, Ministry of Agriculture and Forestry, 55200 Atakum, Samsun Turkey; 5 Department of Biology, Faculty of Science, Ondokuz Mayis University, 55270 Atakum, Samsun Turkey

**Keywords:** Canine parvovirus, Red foxes, Isolation, PCR, Phylogeny

## Abstract

**Background:**

The canine parvovirus, with its many variants, is responsible for a pivotal and common viral infection affecting millions of dogs and other carnivore species worldwide, particularly the wild ones, which are considered as the main reservoir hosts. To that end, this study investigated the presence of canine parvovirus (CPV) in red foxes (*Vulpes vulpes*) living in wild habitats of several regions of Turkey.

**Methods:**

We randomly collected 630 archival fox stool specimens from rural areas of 22 provinces and used real-time PCR to detect CPV.

**Results:**

Two of the 630 (0.3%) stool samples were positive for CPV-DNA, named Tr-Fox/128(Aydın) and Tr-Fox/159(Manisa). We attempted to isolate the virus in a MDCK cell line, and cytopathic effects were observed four days post-inoculation. Three regions corresponding to the CPV capsid protein VP2 gene from extracted DNA of positive samples were amplified by conventional PCR, and the products were visualised, purified, and Sanger sequenced. Three overlapping DNA raw sequence fragments, were read, assembled, and aligned to obtain approximately 1.5 kb-long regions that cover most of the VP2 gene, then deposited in GenBank. After comparing the isolates with parvovirus sequences data of domestic and wild carnivores by BLAST processing, our isolates' similarity rate with each other was 99.40%, with base differences in 9 nucleotide positions. They were classified as 2b variant closely related to isolates from dogs in Turkey, Egypt, Iraq, Italy, Thailand, and China.

**Conclusion:**

This study presents evidence of interspecies transmission of CPV, of which there are no reports on prevalence in wildlife carnivores of our country. Identification of CPV in red foxes threatens local and hunting dogs, which may contract the infection or disseminate it to other wild animal species or vice-versa.

**Supplementary Information:**

The online version contains supplementary material available at 10.1186/s12985-023-01988-2.

## Background

*Canine parvovirus* (CPV), also known as *Carnivore protoparvovirus-1*, is a highly transmissible pathogen of dogs and a leading cause of severe gastroenteric disease, with high morbidity, and it is common all over the world [1]. The etiological agent is a member of the genus *Protoparvovirus*, which belongs to subfamily *Parvovirinae* of the *Parvoviridae* family [2]. CPV has a small, icosahedral capsid, non-enveloped DNA genome encompassing nearly 5000 nucleotides (nt) that encode three structural capsid proteins and two non-structural (NS) proteins (VP1, VP2, VP3, NS1 and NS2). VP2 is the immunodominant structural protein. This major capsid protein presents antigenic sites and is responsible for the interactions between virion and cellular receptors [3, 4].

The history of CPV started during the early 1970s, when a novel parvovirus was identified and isolated in canine and feline cell cultures. It was named CPV-2 to distinguish it from CPV-1 (canine minute virus). The two parvoviruses are antigenically unrelated, with notable differences [3]. Three antigenic variant strains of CPV-2 are recognized—2a, 2b, and 2c—based on at least five or six VP2 amino acid substitutions from the CPV-2 original virus, which is no longer present in canine populations, although its variants are frequently detected [5]. CPV-2c is predominant in Europe, North America, and South America [6–9], whereas CPV-2a and CPV-2b are generally reported from Asia and Africa [5, 10, 11]. More recently, CPV-2c and CPV-2b has been observed in Australia [12, 13].

CPV has a broad host range that includes dogs (the primary host) as well as other domestic and wild animals, especially members of order *Carnivora* such as foxes and wolves from family *Canidae*; cats, civet cats, and leopards from family *Felidae*; ferrets and minks from family *Mustelidae*; and raccoons from family *Procyronidae* [14]. Like all parvoviruses, CPV is highly stable to external environmental conditions and can persist in domestic carnivorans populations' habitats for five months or longer because of fecal–oral transmission. This route may also spread the infection to wild canids [14]. On the other hand, wild canids, particularly urban red foxes (*Vulpes vulpes*), can also act as a reservoir of CPV infection and disseminate the disease to domestic canine populations [2].

Parvoviruses invade the pharynx first and then multiply in the lymphocytes and pass into the bloodstream within a few days of infection to reach organs with rapidly-dividing cells, such as intestines and bone marrow where white blood cells can be infected, resulting in profound leucopenia. Then over the next three to five days, these viruses cause viremia that enables them to replicate in the muscles of the heart, which later may lead to myocarditis, especially in puppies born to unvaccinated parents[1]. Also, parvoviruses form large eosinophilic intranuclear inclusion bodies, which may cause significant damage, especially in the gastrointestinal tract where these viruses get inside into their germinal epithelium leading to diarrhea as they infect the crypts of Lieberkuhn causing villous collapse and failure of nutrients absorption [1]. CPV infection has various clinical manifestations, particularly in dogs younger than six months. Severe, malodorous bloody diarrhea containing mucus is the predominant symptom of this virus infection, followed by vomiting, anorexia, lethargy, and fever [4, 15].

Serological and molecular investigations of CPV in different parts of the globe show high prevalence in several species of domestic, captive, and free-ranging wild carnivores that can reach 76% [16–18]. Although this virus has been documented in cats and dogs in different regions of Turkey by molecular and serological techniques [19–22], we could not find any notification of CPV in wildlife species. Due to the infectivity of this pathogen and to identify reservoir species and carrier hosts, we sought to identify CPV strains circulating in red foxes (*Vulpes vulpes*) living in wild habitats of several regions in Turkey, along with isolation and molecular characterization to elucidate genetic relationship with other strains that can infect domestic animals.

## Methods

### Study area

**Fig. 1:**
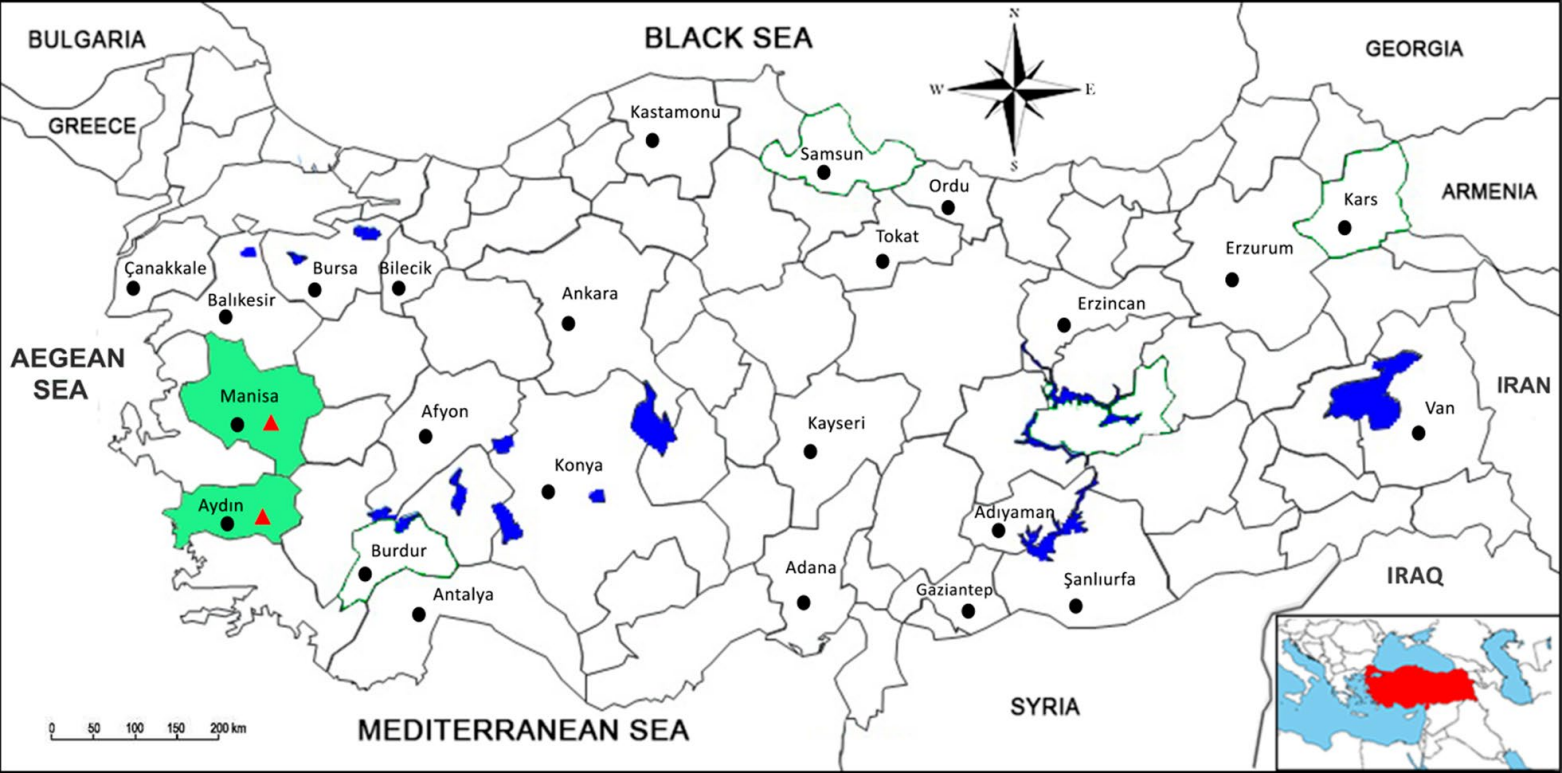
The map of Turkey shows the geographical locations of provinces where stool samples were collected.  = provinces where fox stools were collected.  = provinces where CPV-positive fox stools were detected

Turkey lies between longitudes 36–42° North and 26–45° East. This study used 630 archived stool specimens collected from red foxes living in the wild habitat of 22 provinces of all seven geographical regions of Turkey, as Fig. 1 depicts. Fox stool specimens were randomly collected from distinctive suitable locations in rural areas, including villages and fields where fox holes were at least 1 km from each other.

Gürler et al. [23] reported morphological and macroscopic criteria for distinguishing red foxes' feces, including size, sharpness, odor, and food residues such as feathers, hairs, and fruits. We used their criteria for samples. We collected freshly voided and old dried fox stools from the ground with disposable gloves, placed the samples into sterile containers, and transmitted them to the lab under cold chain specifications, stored at − 80 °C. We made up 63 pools containing 10 stool samples each to facilitate the molecular investigation of CPV.

### Real-time PCR assay

DNA was extracted from each stool pool using PureLink Genomic DNA Mini Kit (Invitrogen, Cat.No.K1820-02,USA) according to the manufacturer's instructions. A real-time PCR test was performed in a CFX Connect real-time PCR machine (Biorad, USA) with primers and probe previously described by Decaro et al. [24], as detailed in Table 1. The reactions for real-time PCR were conducted on 25 μl final volume containing 5 μl of template DNA, 0.75 μl of dNTP, 2.5 μl of 10X buffer, 500 nM of each primer, 300 nM of the probe, 1 μl of Taq polymerase, 2.5 μl of MgCl_2,_ and 10 μl of RNAse-free water. PCR test conditions were adjusted as follows: 1 min at 95 °C, followed by 40 cycles at 95 °C for 15 s, 52 °C for 30 s, and 72 °C for 60 s. Parvovirus vaccine (Novibac, UK) was a positive control in all PCR assays.

**Table 1:** Primers and probe were used for CPV PCR testing and sequencing

Primers & probe	Sequences (5′ 3′)	Amplicon (bp)	References
CPV-for	AAACAGGAATTAACTATACTAATATATTTA	93	[24]
CPV-rev	AAATTTGACCATTTGGATAAACT		
CPV-Pb	FAM–TGGTCCTTTAACTGCATTAAATAATGTACC-TAMRA		
Pabs	GAAGAGTGGTTGTAAATAATT	681	[25]
Pabas	CCTATATAACCAAAGTTAGTAC		
Hfor	CAGGTGATGAATTTGCTACA-3	630	[3]
Hrev	CATTTGGATAAACTGGTGGT-3		
555for	CAGGAAGATATCCAGAAGGA	583	[3]
555rev	GGTGCTAGTTGATATGTAATAAACA		

*for* Forward; *rev* Reverse; *Pb* Probe; *bp* Base pair

### Cell culture, virus isolation, and confirmation with PCR assays

Madin Darby canine kidney (MDCK) cell lines were utilized to isolate CPV from stool samples. Briefly, MDCK cells were grown at 37 °C with 5% CO_2_ in Eagle's Minimal Essential Medium (EMEM, Sigma, UK) supplemented with 10% fetal calf serum (FCS, Sigma, UK) and 1% antibiotics (10,000 U/mL) (penicillin–streptomycin, Gibco, USA) and were stored at − 80 °C. The cell line and all chemicals used in cell culture were checked for contamination with non-cytopathic pestivirus by conventional PCR test and found to be negative.

Stool samples for virus isolation were prepared using the traditional method. Briefly, 10% suspensions of stool samples in PBS were shaken vigorously through tissuelyser LT (Qiagen, Germany) with sterile beads and centrifuged at 3000 rpm for 30 min at 4 °C. The supernatant was then sterilized by passing through a 0.22 nm disposable filter (Millipore, USA) during inoculation into cell lines. Virus isolation was attempted from stool samples found to be positive by real-time PCR test; then, consecutive passages were carried out in cell lines. Briefly, 500 µl sterile stool sample supernatants were inoculated into fresh MDCK monolayers after incubation at 37 °C with 5% CO_2_ for 60 min. Following absorption, supernatants were replaced with 5 ml EMEM supplemented with 2% FCS. Cells were kept in a 37 °C incubator containing 5% CO_2_ with daily basis checking using an inverted microscope (Olympus, Japan) to observe any cytopathic effects (CPE). Real-time PCR was used to confirm CPV in infected cell lysates.

### Conventional PCR

Extracted viral DNA from stools was amplified using three primer pairs targeting the VP2 gene of CPV (Table 1). All PCR assays were executed in a 50 μl final volume, divided as 5 μl of template DNA, 1 μl of dNTP, 5 μl of 10 × buffer, 2 μl of each primer, 1 μl of Taq polymerase, 5 μl of MgCl2, and 29 μl of RNAse-free water. PCR conditions were optimized as the same for the three PCR tests and performed as follows: 1 min at 95 °C, followed by 40 cycles at 95 °C for 15 s, 52 °C for 30 s, and 72 °C for 60 s. Then 10 μl of each PCR product was loaded on a 2% agarose gel stained with ethidium bromide, ran at 100 V for 35 min, then visualized using the Quantum gel imaging and documentation system (Vilber Lourmat, Collegien, France) to visualize the bands corresponding to 681 bp, 630 bp, and 583 bp of CPV.

### Partial sequencing and phylogenetic analysis

PCR amplicons were purified using a QIAquick PCR purification kit (Qiagen, Germany) and then Sanger sequencing was done (RefGen Biotechnology, Ankara, Turkey) (http://www.refgen.com). The three PCR amplicons' raw sequences were assembled, aligned using the Bioedit version 7.2.5 program (Informer Technologies) and deposited into GenBank [26]. After BLAST analysis, we compared our deposited sequences with 63 representative strains' sequences, including both CPV and *Feline Panleukopenia Virus* (FPLV) isolates of domestic and wild carnivores obtained from GenBank. The phylogenetic tree was constructed based on VP2 gene nucleotide (nt) sequences with the Neighbor-Joining (NJ) method using MEGA X (Molecular Evolutionary Genetics Analysis-MEGA, version 10.0.5) under the Tamura-3 parameter model [26].

## Results

Two of the 63 (3.17%) pooled stool samples were positive after real-time PCR. The corresponding virus-positive pools originated from the Manisa and Aydın provinces of Western Anatolia (Fig. 1). The 20 samples in the two pools were again tested individually by real-time PCR to determine the exact number of fox-positive stools. Only one positive stool sample was detected from each of the two CPV-2-positive pools and labeled as Tr-Fox/128(Aydın) and Tr-Fox/159(Manisa). The total positivity rate of CPV-2 in fox stools was 0.3% (2/630).

**Fig. 2:**
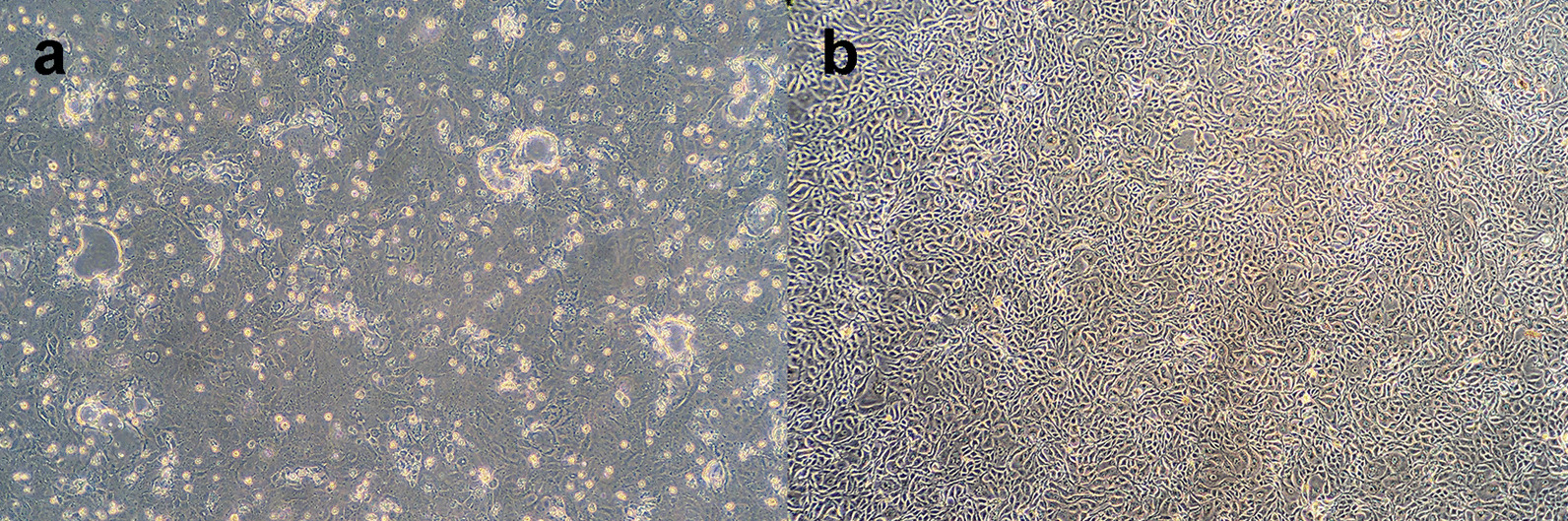
Photos taken from an inverted microscope show the CPV growth in the MDCK cell line four days post-inoculation. **a**: CPE changes of Tr-Fox/128 (MW259073.1) observed after inoculation in MDCK cells, including rounding, clumping, and detachment of cells from the monolayer surface. **b**: Cell control of MDCK cell line (magnification 40×)

To isolate the virus, supernatants of the two positive stool samples were inoculated in the MDCK cell line and subjected to at least three passages. Only Tr-Fox/128 showed CPE. All CPE changes, including rounding, granulation, clumping, and detachment from the monolayer surface, were observed four days post-infection (Fig. 2). We did not observe CPE changes for the virus isolation attempt from the Tr-Fox/159, positive sample despite identification of CPV using PCR.

Three partial genes of VP2 from extracted DNA of positive samples were amplified by conventional PCR test using specific primers for this VP2 gene that encodes the capsid protein of CPV. Three VP2 gene PCR products, corresponding to 681 bp, 630 bp, and 583 bp bands, were visible after gel running for both isolates. After the sequences of the three overlapping DNA fragments that were aligned using the MEGA X program, a consensus sequence emerged for approximately 1.5-kb long regions that cover most of the VP2 gene. These sequences were deposited in GenBank under accession numbers MW259073.1 for Tr-Fox/128 and MW259074.1 for Tr-Fox/159 (see Additional file 1).

**Fig. 3:**
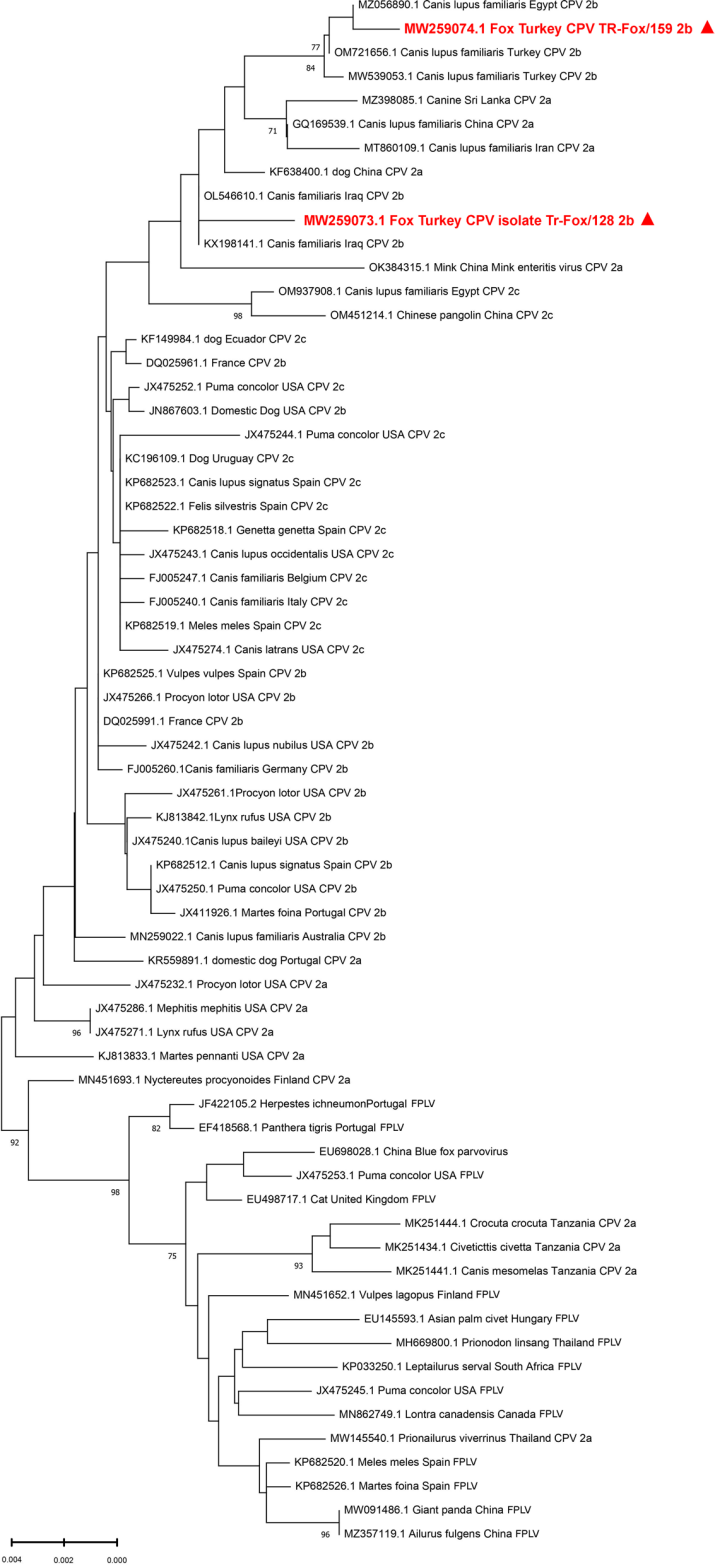
Phylogenetic tree constructed using the maximum-likelihood method from complete VP2 gene nucleotide sequences of CPV and assessed using 1000 bootstrap replications. Red triangles represent our isolates. Others are relative CPV and FPLV strains or isolates labeled with their GenBank accession number, animal host, country and virus name

We compared the 1512-bp-long sequences to parvovirus sequence data reported in GenBank by BLAST analysis to create a phylogenetic tree (Fig. 3). Tr-Fox/128 isolate with GenBank accession number MW259073.1 was similar to sequences of strains isolated from dogs (*Canis lupus familiaris*) with a 99.74% similarity to isolates (KX198141.1 and OL546610.1) from Iraq, 99.67% similarity to isolate (OL546608.1) from Iraq, 99.60% similarity to isolate (MN104213.1) from Italy, and 99.60% similarity to isolate (KR002798.1) from China. Conversely, the sequence data of Tr-Fox/159 isolate with GenBank accession number MW259074.1 was similar to sequences of strains isolated from dogs (*Canis lupus familiaris*), with a 99.87% similarity to isolates (MZ056890.1, MZ056888.1, and MZ056882.1) from Egypt, 99.80% similarity to isolate (OM721656.1) from Turkey, and 99.74% similarity to sequence data of isolate (KP715703.1) from Thailand.

The similarity index rate among our Tr-Fox/128 and Tr-Fox/159 isolates was 99.40%. They differ in nine nucleotide positions. Nevertheless, only one amino acid (aa) difference was found among them. Because self-comparison of the isolates revealed that phenylalanine (F) was at the 267th aa position in the Tr-Fox/128 isolate, but it had been converted to tyrosine (Y) in the Tr-Fox/159 isolate. There are no studies on CPV-2 in wildlife animal species in Turkey, so we compared the isolates to isolates from domestic animals in Turkey (Table 2).

**Table 2:** Variable nucleotides and amino acid positions in VP2 gene sequences from this study and other Turkish isolates

Isolates	Nucleotide positions												Amino acid position
	303	384	534	732	750	756	**800**	855	948	1251	1335	1659	**267**
MW259073.1 TR 128	C	A	T	T	A	A	**T**	A	G	C	T	T	**F**
MW259074.1 TR 159	T	G	C	C	G	G	**A**	A	G	C	C	C	**Y**
KX268119.1 TR	T	A	T	T	G	A	**T**	G	A	T	T	T	**F**
KX268109.1 TR	T	A	T	T	G	A	**T**	G	A	T	T	T	**F**
OM721656.1 TR	T	A	C	C	G	G	**A**	G	G	T	C	C	**Y**
OM721655.1 TR	T	A	C	C	G	G	**A**	G	A	T	T	C	**Y**
MW539053.1 TR	C	A	C	C	G	G	**A**	G	G	T	C	C	**Y**
MT952859.1 TR	T	A	T	C	G	A	**A**	G	A	T	T	T	**Y**

We cited the amino acid type at the 426 position of the VP2 capsid protein because serological tests were not performed. According to Miranda et al. [27] contains asparagine-Asn (N) in vaiant 2a, aspartic acid-Asp (D) in vaiant 2b, and glutamic acid-Glu (E) in vaiant 2c. The two isolates found in this study were sorted with the 2b variant type because they each have aspartic acid (D) in position 426 of the amino acid sequence of VP2 capsid protein.

## Discussion

Canine parvovirus infection is one of the most pivotal and common viral diseases worldwide, affecting dogs and wild carnivores. In recent years, many studies have attempted to detect the virus, especially in wild animals. Molecular studies on dogs have found three variants of CPV in local circulation in different regions of Turkey [20, 21, 28–32]. However, no reports of any types of CPV were found in wild carnivores, such as foxes, wolves, and coyotes, from our country. This study is the first to identify the virus in a wild carnivore species, the red fox. Variants 2a and 2b have been predominantly identified among domestic animals in Turkey. The recent detection of the 2b variant in dogs (GenBank accession number MW539053.1) from Izmir province in the Aegean region of Turkey close to the provinces where our positive samples were found suggests possible virus transmission from domestic dogs to wild animals in this study. Domestic and rural dogs frequently interact with wild carnivores as contenders, prey, or predators. The high population densities of domesticated canines worldwide impact the habitats and activity patterns of wild canines [16].

The bridge that domestic canids create with sylvatic environments facilitates transmission of pathogens, including CPV, to naive populations of wildlife species, causing epidemics, especially near protected areas [16, 33]. In this context, we noted a positive correlation in Italy between CPV variants from domestic dogs and wolves, supporting the significance of the reservoir role that domestic dogs play in transmitting this viral etiological agent [34]. Similar findings are from a study conducted in the USA that detected CPV-2c and CPV-2b in stool samples of the gray wolf (*Canis lupus*), coyote (*Canis latrans*), and puma (*Puma concolor*), whereas all three variants (CPV-2a, CPV-2b, and CPV-2c) were detected among commingled domestic dogs [27, 35]. Furthermore, in other countries known to have a wildlife habitat like Namibia, CPV-2b was the major circulating variant among both wild carnivores and dogs. CPV-2b was also identified in bat-eared foxes (*Otocyon megalotis*) and cheetahs (*Acinonyx jubatus*), which indicates a possible positive correlation [27, 36]. Additionally, the CPV-2a local variant in dogs in Germany was also detected in Siberian tigers (*Panthera tigris tigris*) of the same country, which reinforces the correlation between the circulation of different antigenic variants of CPV and their overcoming of the interspecies barrier [27, 36].

Other wildlife surveillance for CPV identified different circulating variants among populations of wild carnivores of various species. For example, a phylogenetic study in Portugal detected CPV-2b and CPV-2a in wolves (*Canis lupus*) and CPV-2c in martens (*Martes foina*) [27]. Similar to our outcomes, Spera et al. [18] detected CPV-2 in a crab-eating fox (*Cerdocyon thous*) and classified the causative virus as a 2b variant. A more recent report by Ndiana et al. [37] detected parvovirus DNA in 34 out of 297 wild animals (red foxes, wolves, Eurasian badgers, and martens). Their molecular characterization revealed coinfections of CPV-2b/2c and CPV-2b/FPV in six wolves and two badgers. The study also detected CPV-2a in four wolves and one badger, CPV-2b in 11 wolves, five badgers, and one marten, and CPV-2c in 10 wolves and one badger. Our findings highlight the importance of the 2b variant because it is now most frequently seen in domestic and wild carnivores. Therefore, this variant may pose a future threat if mutations increase virulence, a possibility suggested by the frequently observed interspecies transmission.

## Conclusion

This study is the first to reveal parvovirus infection in red foxes in Turkey. Interspecies transmission might arise from the consumption of infected meat or bones in meal remnants from common dogs or through the fecal–oral route by close contact between infected domestic animals and wild animals. This is more likely in rural settlements, where the virus is capable of jumping species barriers to spread between domestic and hunting dogs. Further comprehensive molecular epidemiological studies of wildlife in Turkey will elucidate the genetic diversity of CPV variants, which may determine vaccination strategies in various carnivore species.

## Supplementary Information

**Additional file 1.** VP2 gene partial sequence of our both isolates.

## Data Availability

The data presented in the study were deposited in the NCBI repository, with accession numbers MW259073.1 and MW259074.1. VP2 gene partial sequence of our both isolates are available as a supplementary file (see Additional file 1).
