# Supplementary material for: First isolation and molecular characterization of canine parvovirus-type 2b (CPV-2b) from red foxes (Vulpes vulpes) living in the wild habitat of Turkey
Source: Virol J. 2023 Feb 11;20:27. doi: 10.1186/s12985-023-01988-2 (PMC9921602; doi:10.1186/s12985-023-01988-2)
Supplement: Supplementary file 1 — Additional file 1. VP2 gene partial sequence of our both isolates. [file 12985_2023_1988_MOESM1_ESM.docx]

**>MW259073.1 Carnivore protoparvovirus 1 isolate Tr-Fox/128 capsid protein (VP2) gene, partial cds**

AGAGTGGTTGTAAATAATTTGGATAAAACTGCAGTTAACGGAAACATGGCTTTAGATGATACCCATGCACAAATTGTAACACCTTGGTCATTGGTTGATGCAAATGCTTGGGGAGTTTGGTTTAATCCAGGAGATTGGCAACTAATTGTTAATACTATGAGTGAGTTGCATTTAGTTAGTTTTGAACAAGAAATTTTTAATGTTGTTTTAAAGACTGTTTCAGAATCTGCTACTCAGCCACCAACTAAAGTTTATAATAATGATTTAACTGCATCATTGATGGTTGCATTAGATAGTAATAATACTATGCCATTTACTCCAGCAGCTATGAGATCTGAGACATTGGGTTTTTATCCATGGAAACCAACCATACCAACTCCATGGAGATATTATTTTCAATGGGATAGAACATTAATACCATCTCATACTGGAACTAGTGGCACACCAACAAATATATACCATGGTACAGATCCAGATGATGTTCAATTTTATACTATTGAAAATTCTGTACCAGTACACTTACTAAGAACAGGTGATGAATTTGCTACAGGAACATTTTTTTTTGATTGTAAACCATGTAGACTAACACATACATGGCAAACAAATAGAGCATTAGGCTTACCACCATTTCTAAATTCTTTGCCTCAAGCTGAAGGAGGTACTAACTTTGGTTATATAGGAGTTCAACAAGATAAAAGACGTGGTGTGACTCAAATGGGAAATACAAACATTATTACTGAAGCTACTATTATGAGACCAGCTGAGGTTGGTTATAGTGCACCATATTATTCTTTTGAGGCGTCTACACAAGGGCCATTTAAAACACCTATTGCAGCAGGACGGGGGGGAGCGCAAACAGATGAAAATCAAGCAGCAGATGGTGATCCAAGATATGCATTTGGTAGACAACATGGTCAAAAAACTACCACAACAGGAGAAACACCTGAGAGATTTACATATATAGCACATCAAGATACAGGAAGATATCCAGAAGGAGATTGGATTCAAAACATTAACTTTAACCTTCCTGTAACAGATGATAATGTATTGCTACCAACAGATCCAATTGGAGGTAAAGCAGGAATTAACTATACTAATATATTTAATACTTATGGTCCTTTAACTGCATTAAATAATGTACCACCAGTTTATCCAAATGGTCAAATTTGGGATAAAGAATTTGATACTGACTTAAAACCAAGACTTCATGTAAATGCACCATTTGTTTGTCAAAATAATTGTCCTGGTCAATTATTTGTAAAAGTTGCGCCTAATTTAACAAATGAATATGATCCTGATGCATCTGCTAATATGTCAAGAATTGTAACTTACTCAGATTTTTGGTGGAAAGGTAAATTAGTATTTAAAGCTAAACTAAGAGCCTCTCATACTTGGAATCCAATTCAACAAATGAGTATTAATGTAGATAACCAATTTAACTATGTACCAAGTAATATTGGAGGTATGAAAATTGTATATGAAAAATCTCAACTAGCACCTAGAAAATTATAT

**>MW259074.1 Carnivore protoparvovirus 1 isolate Tr-Fox/159 capsid protein (VP2) gene, partial cds**

AGAGTGGTTGTAAATAATTTGGATAAAACTGCAGTTAACGGAAACATGGCTTTAGATGATACTCATGCACAAATTGTAACACCTTGGTCATTGGTTGATGCAAATGCTTGGGGAGTTTGGTTTAATCCAGGAGATTGGCAACTGATTGTTAATACTATGAGTGAGTTGCATTTAGTTAGTTTTGAACAAGAAATTTTTAATGTTGTTTTAAAGACTGTTTCAGAATCTGCTACTCAGCCACCAACTAAAGTTTATAATAATGATTTAACTGCATCATTGATGGTTGCATTAGACAGTAATAATACTATGCCATTTACTCCAGCAGCTATGAGATCTGAGACATTGGGTTTTTATCCATGGAAACCAACCATACCAACTCCATGGAGATATTATTTTCAATGGGATAGAACATTAATACCATCTCATACTGGAACTAGTGGCACACCAACAAATATATACCATGGTACAGATCCAGATGATGTTCAATTTTACACTATTGAAAATTCTGTGCCAGTGCACTTACTAAGAACAGGTGATGAATTTGCTACAGGAACATTTTATTTTGATTGTAAACCATGTAGACTAACACATACATGGCAAACAAATAGAGCATTAGGCTTACCACCATTTCTAAATTCTTTGCCTCAAGCTGAAGGAGGTACTAACTTTGGTTATATAGGAGTTCAACAAGATAAAAGACGTGGTGTGACTCAAATGGGAAATACAAACATTATTACTGAAGCTACTATTATGAGACCAGCTGAGGTTGGTTATAGTGCACCATATTATTCTTTTGAGGCGTCTACACAAGGGCCATTTAAAACACCTATTGCAGCAGGACGGGGGGGAGCGCAAACAGATGAAAATCAAGCAGCAGATGGTGATCCAAGATATGCATTTGGTAGACAACATGGTCAAAAAACTACCACAACAGGAGAAACACCTGAGAGATTTACATATATAGCACATCAAGATACAGGAAGATATCCAGAAGGAGATTGGATTCAAAACATTAACTTTAACCTTCCTGTAACAGATGATAATGTATTGCTACCAACAGATCCAATTGGAGGTAAAGCAGGAATTAACTATACCAATATATTTAATACTTATGGTCCTTTAACTGCATTAAATAATGTACCACCAGTTTATCCAAATGGTCAAATTTGGGATAAAGAATTTGATACTGACTTAAAACCAAGACTTCATGTAAATGCACCATTTGTTTGTCAAAATAATTGTCCTGGTCAATTATTTGTAAAAGTTGCGCCTAATTTAACAAATGAATATGATCCTGATGCATCTGCTAATATGTCAAGAATTGTAACTTACTCAGATTTTTGGTGGAAAGGTAAATTAGTATTTAAAGCTAAACTAAGAGCCTCTCATACTTGGAATCCAATTCAACAAATGAGTATCAATGTAGATAACCAATTTAACTATGTACCAAGTAATATTGGAGGTATGAAAATTGTATATGAAAAATCTCAACTAGCACCTAGAAAATTATAT
